# Novel potential metabolic biomarker panel for early detection of severe COVID-19 using full-spectrum metabolome and whole-transcriptome analyses

**DOI:** 10.1038/s41392-022-00976-2

**Published:** 2022-04-22

**Authors:** Zhi-Bin Li, Jun Liu, Shan-Qiang Zhang, Yi Yu, Hong-Feng Liang, Qi-Qi Lu, Jing Chen, Yu-Shuai Han, Fan Zhang, Ji-Cheng Li

**Affiliations:** 1 The Central Laboratory, Yangjiang People’s Hospital, 529500 Yangjiang, China; 2 Medical Research Center, Yue Bei People’s Hospital, Shantou University Medical College, 512025 Shaoguan, China; 3 Medical School, Hubei Minzu University, 445000 Enshi, China; 4 Department of Histology and Embryology, Shaoguan University School of Medicine, 512025 Shaoguan, China; 5 Institute of Cell Biology, Zhejiang University School of Medicine, 310058 Hangzhou, China

**Keywords:** Predictive markers, Infectious diseases

**Dear Editor**,

The mortality rate of severe coronavirus disease 2019 (COVID-19) is high, and there is no universally accepted method to predict its occurrence. COVID-19 pneumonia can be classified into four categories, such as, asymptomatic, mild, moderate, and severe. According to Wu et al.^1^ data regarding COVID-19 patients in mainland China showed that among 44,415 patients who suffered from COVID-19, 36,160 patients had mild COVID-19, while the remaining 8255 patients (about 19%) developed severe COVID-19, leading to respiratory failure, acute respiratory distress syndrome (ARDS), multi-organ failure, and even death. At present, the diagnosis of patients with severe COVID-19 relies primarily on clinical symptoms. However, once these symptoms including dyspnea, reduced blood oxygen levels, and severe acidosis are present, the condition may deteriorate rapidly and can lead to death. Therefore, seeking biomarkers for early detection of COVID-19 can be of great significance to improve the prognosis of critically ill patients with COVID-19.

This study adopted liquid chromatography–tandem mass spectrometry to screen differentially expressed metabolites in the plasma of mild COVID-19 patients, severe COVID-19 patients, community-acquired pneumonia (CAP) patients and healthy control subjects. Further based on the identified metabolites, the main metabolic pathways involved were analyzed. A model for identifying severe COVID-19 was constructed and validated using the least absolute shrinkage and selection operator (LASSO) method. Subsequently, differentially expressed RNAs were screened in peripheral blood mononuclear cells (PBMCs) with whole-transcriptome sequencing, followed by metabolome–transcriptome correlation analysis. Detailed description of the methods is provided in Supplementary material.

In this study, a total of 128 samples were selected and divided into four groups for metabolic studies (Supplementary Table S1). Metabolic analysis based on a ultra-high performance liquid chromatography-MS/MS (UPLC-MS/MS) platform with a wide range of targeted metabolomic techniques detected a total of 600 differential metabolites. Orthogonal partial least squares discriminant analysis (OPLS-DA) revealed a clear differentiation between the severe COVID-19 group and the healthy control group, the severe COVID-19 group and the CAP group, and the severe COVID-19 group and the mild COVID-19 group (Supplementary Fig. S1a–c). In addition, the results of principal component analysis (PCA) (Supplementary Fig. S1d) and 3D-PCA (Supplementary Fig. S1e) showed better differentiation among the groups. With a fold-change ≥2 or ≤0.5 and VIP ≥ 1 as screening criteria, 55 differential metabolites were downregulated and 58 differential metabolites were upregulated in the severe COVID-19 group, compared with the healthy control group (Fig. 1a). In addition, 79 differential metabolites were downregulated and 44 differential metabolites were upregulated in the severe COVID-19 group, compared with the CAP group (Fig. 1b). Besides, 26 differential metabolites were downregulated and 23 differential metabolites were upregulated in the severe COVID-19 group, compared with the mild COVID-19 group (Fig. 1c). A total of 32 differentially expressed metabolites in patients with severe COVID-19 were obtained from the comparison across four groups (Fig. 1d).

**Fig. 1:**
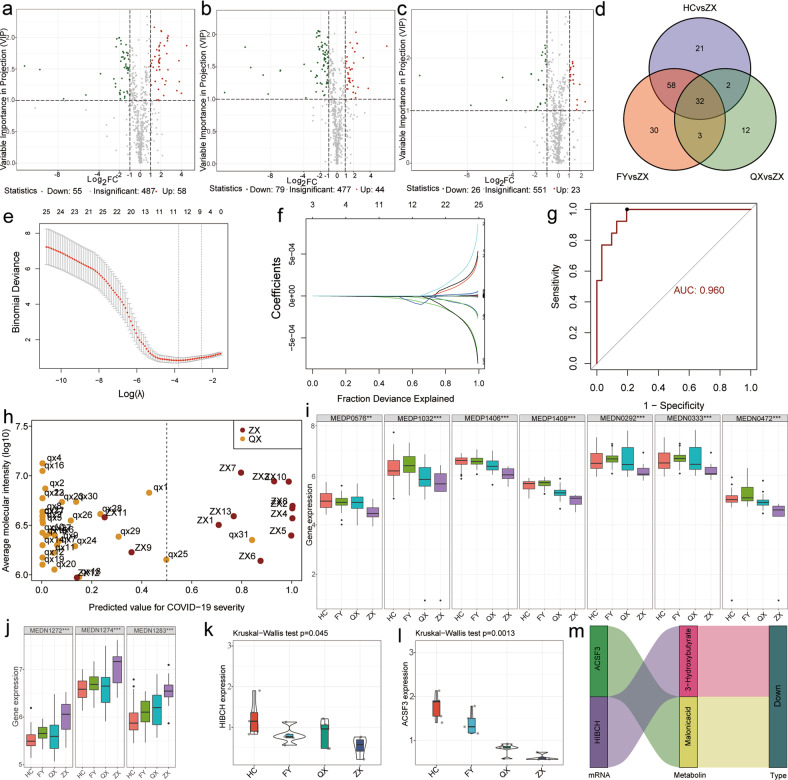
Novel potential metabolic biomarker panel for early detection of severe COVID-19. HC, healthy controls. FY, community-acquired pneumonia. QX, mild COVID-19 patients. ZX, severe COVID-19 patients. Differences in metabolites between severe COVID-19 patients and healthy controls (**a**), CAP (**b**), and mild COVID-19 (**c**) are illustrated by volcano plots. **d** Venn diagram analysis showing shared differentially expressed metabolites in the severe COVID-19 group compared with the other three groups. **e**, **f** Metabolites presenting specificity in severe COVID-19 patients screened by LASSO regression. **g** Receiver operating characteristics analysis demonstrating the diagnostic value of this diagnostic panel in severe COVID-19 patients and mild COVID-19 patients. **h** Performance of this diagnostic model in differentiating between severe COVID-19 patients and mild COVID-19 patients. **i**, **j** Expression levels of the ten differential metabolic markers in the four groups. **k** Expression of *HIBCH* in the four groups. **l** Expression of *ACSF3* in the four groups. **m** Potential regulatory relationship between *HIBCH* and *ACSF3*

Subsequently, ten differential metabolites that could differentiate between severe COVID-19 patients and mild COVID-19 patients were identified by LASSO regression (Fig. 1e, f). A diagnostic model based on the ten differential metabolites was constructed by stepwise logistic regression (Fig. 1g). The ROC curve analysis evaluated the diagnostic value of the model (with an area of 0.96 under the curve) in distinguishing between severe COVID-19 patients and mild COVID-19 patients (Fig. 1g). Statistical data revealed that that the model could accurately distinguish between 30 mild COVID-19 patients and severe COVID-19 patients (Fig. 1h). To further explore the possible biological significance of differentially expressed molecules, the ten differential metabolites were analyzed. Interestingly, eight of these ten differential metabolic markers were lipids or closely related to lipid metabolism (Supplementary Table S2). In addition, three of these eight markers were glycerophospholipids, two were fatty acyls, and three (3-hydroxybutyric acid, malonic acid, and 1-methyluronic acid) were closely related to lipid metabolism. These three glycerophospholipids were upregulated (Fig. 1j) in the COVID-19 group, and their upregulation was more significant in the severe COVID-19 group (*p* < 0.01). Fatty acy l, 3-hydroxybutyric acid, malonic acid, and 1-methyluronic acid were downregulated (Fig. 1i) in the COVID-19 group (*p* < 0.01) and were more significantly downregulated in the severe COVID-19 group (*p* < 0.01). Our findings suggested that abnormalities in glycerophospholipid metabolism may be an important intrinsic attribute in COVID-19.

Interestingly, this finding is also supported by other researchers. For example, Shen et al.^2^ found abnormal expression of several apolipoproteins and abnormal expression of sphingolipids and glycerophospholipids in patients with COVID-19. The introduction of a CAP control group in the present study further confirmed the high specificity of abnormal glycerophospholipid metabolism in patients with COVID-19, compared with patients with other lung diseases. Furthermore, the comparison between mild COVID-19 patients and severe COVID-19 patients revealed more severe abnormalities in glycerophospholipid metabolism in patients with severe COVID-19 (Fig. 1i, j). Moreover, in terms of research technology, this study adopted a full-spectrum metabolic technology (see Supplementary material for details). We identified and compared differentially expressed non-lipid and lipid metabolites, and screened them through machine learning methods. We found that among differential metabolites, lipids, especially glycerophospholipid had more significant differences. This further indicated the characteristic of abnormal lipid metabolism in COVID-19. In addition, we performed transcription–metabolism association analysis with differential metabolites and predicted the upstream target genes that could lead to abnormal lipid metabolism inorder to evaluate the possible mechanism involving abnormal lipid metabolism and the possible relationship between the lipid metabolism and immune regulation and inflammation.

Several studies have demonstrated the close correlation between COVID-19 severity and inflammatory cytokine storm and the interactions between metabolites and immune cells. Therefore, we recruited a new cohort including 19 PBMCs samples (four severe COVID-19 cases, five mild COVID-19 cases, five CAP cases, and five healthy controls, Supplementary Table S1) were selected for the whole-transcriptome high-throughput sequencing covering circular RNAs (circRNAs), long-noncoding RNAs (lncRNAs), microRNAs (miRNAs), and messenger RNAs (mRNAs) data (Supplementary Figs. S2–S4). Detailed description of the differentially expressed RNAs is provided in Supplementary material. PBMCs whole-transcriptome analysis identified changes in various molecules within immune cells and enrichment of related pathways (Supplementary Fig. S6). Gene set variation analysis (GSVA) was performed separately in the severe COVID-19 group and the other groups (Supplementary Fig. S7) to further explore potential mechanisms in the pathogenesis of severe COVID-19. It is worth noting that the results indicated that fatty acid metabolism was significantly inhibited at the transcriptome level (Supplementary Fig. S7).

Moreover, a metabolome–transcriptome association analysis predicted possible upstream target genes for aberrant metabolite expression, and the scope of these genes was narrowed down by taking intersections with genes differentially expressed identified by the whole-transcriptome sequencing (Supplementary Fig. S2–6). To explore the effect of the altered transcripts and the production pathways of ten differential metabolite markers were analyzed to elucidate the relevance of *HIBCH* and *ACSF3* genes for the production of 3-hydroxybutyrate and malonic acid, respectively. Subsequently, the expression levels of these genes in the four groups indicated the consistency of the expression trends of these genes with those of 3-hydroxybutyrate and malonic acid in the four groups (Fig. 1k, l). Furthermore, the regulatory roles of *HIBCH* and *ACSF3* on 3-hydroxybutyrate and malonic acid, respectively, were demonstrated by Sankey diagrams (Fig. 1m). *ACSF3* encodes a member of the acyl-coenzyme A synthetase family. It also activates fatty acids by catalyzing the formation of thioester bonds between fatty acids and coenzyme A. The encoded protein, localized to mitochondria, is highly specific for malonic and methylmalonic acids and has malonyl coenzyme A synthase activity.^3^ However, these two genes have rarely been reported in COVID-19 studies; therefore, further research is necessary.

Molecular biology studies have reported that abnormal lipid metabolism is closely associated with COVID-19. It is generally believed that patients with higher lipid levels are more likely to develop severe COVID-19 and inflammatory disease. Interestingly, our study showed that in the absence of significant difference in the baseline data such as, age and blood lipid levels between the disease group and the control group (Supplementary Table 3), the COVID-19 group had characteristic changes in lipid metabolism. This showed that there may be a more complex relationship between lipid metabolism and COVID-19, that need to be evaluated in the future study. Lysophospholipids regulate the modulation functions of immune cells and inflammation.^4^ In addition, lipids, mainly phospholipids and phosphatidylcholine, are important raw materials for the synthesis of lungsurface-active substances. The dysregulation of surfactant membranes is directly related to the onset and progression of ARDS.^5^ The integration of the results in this study with the existing literature.^2,4,5^ led to the following hypothesis: Lipid metabolism remodeling can inhibit the occurrence of cytokine storms. By contrast, for specific lung tissues, lipid metabolism remodeling can affect the secretion of alveolar surface-active substances. The combination of these two factors may have a significant effect on the occurrenceand progression of ARDS and ultimately on the prognosis of patients with severe COVID-19.

This study also has some limitations. Owing to limited amount of blood samples collected from a single patient, this study was unable to detect the metabolome and transcriptome in the same sample. More blood samples need to be collected for future research to address this issue. Also, the sample size need to be expanded in the future study to conduct targeted validation of candidate markers. In addition, more clinical data and the data from other researches need to be analyzed to further validate the findings of this study.

In summary, a panel of ten differential metabolic markers was screened by widely targeted metabolomics analysis. The results revealed that this panel can effectively differentiate severe COVID-19 cases from other cases, and may serve as a potential marker for early detection of severe COVID-19. In addition, the abnormalities in lipid metabolism may be a distinct characteristic of COVID-19, but this warrants further investigation. Besides, developing new diagnostic and therapeutic approaches based on this distinct characteristic is necessary.

## Supplementary information

Sup materials1-Materials and Methods Figures Tables

Sup materials2-ethic approval

Dataset-group name note-normalizeExp

Dataset-figure S2 d normalizeExp-LNCRNA1

Dataset-figure S2 e normalizeExp-LNCRNA2

Dataset-figure S2 f normalizeExp-LNCRNA3

Dataset-figure S3 d normalizeExp-mRNA1

Dataset-figure S3 e normalizeExp-mRNA2.txt

Dataset-figure S3 f normalizeExp-mRNA

Dataset-figure S4 d normalizeExp-CIRC1

Dataset-figure S4 e normalizeExp-CIRC2

Dataset-figure S4 f normalizeExp-CIRC3

Dataset-figure S5 d normalizeExp-MIRNA1

Dataset-figure S5 e normalizeExp-MIRNA2

Dataset-figure S5 f normalizeExp-MIRNA3

## Data Availability

Any data associated with this study are available from the corresponding author upon reasonable request.
