# Supplementary material for: Novel potential metabolic biomarker panel for early detection of severe COVID-19 using full-spectrum metabolome and whole-transcriptome analyses
Source: Signal Transduct Target Ther. 2022 Apr 22;7:129. doi: 10.1038/s41392-022-00976-2 (PMC9026054; doi:10.1038/s41392-022-00976-2)
Supplement: Supplementary file 1 — Sup materials1-Materials and Methods Figures Tables [file 41392_2022_976_MOESM1_ESM.docx]

**Supplemental Material**

**Novel Potential Metabolic BiomarkerPanelfor Early Detection of Severe COVID-19 using Full-Spectrum Metabolome and Whole-Transcriptome Analyses**

Zhi-Bin Li^#,1,2,3,4,5^, Jun Liu^#,2^, Shan-Qiang Zhang^#,2^, YiYu^,1,2,4,5^, Hong-Feng Liang^1^,

Qi-Qi Lu^2^, Jing Chen^1,2,4,5^, Yu-Shuai Han^1,2,4,5^, Fan Zhang^2^, Ji-Cheng Li^1,2,4,5^*

^1^The Central Laboratory, Yangjiang People’s Hospital, Yangjiang 529500, China

^2^Medical Research Center, Yue Bei People’s Hospital, Shantou University Medical College, Shaoguan 512025, China

^3^Medical School,Hubei Minzu University, Enshi 445000, China.

^4^Department of Histology and Embryology, Shaoguan University School of Medicine, Shaoguan 512025, China

^5^Institute of Cell Biology, Zhejiang University School of Medicine, Hangzhou 310058, China

^#^ Contributed equally.

*** Correspondence:**Ji-Cheng Li M.D.
lijichen@zju.edu.cn

Institute of Cell Biology, Zhejiang University School of Medicine, Hangzhou 310058, China

**Supplementary information includes:**

Materials and Methods

Supplementary Figures S1 - S7

Supplementary Tables S1 - S3.

Supplementary References

**Materials and Methods**

***Collection of patients’ blood samples and clinical data***

The study was conducted in accordance with the Declaration of Helsinki,andwas approved by the Ethics Committee of Affiliated Hospital of Zhejiang University (China). Informed consent was obtained from all patients and healthy volunteers. Morning fasting blood samples were collected from all the participants.Specifically, 5 mL of peripheral blood was drawn into a disposable ethylenediaminetetraacetic acid anticoagulated vacuum blood collection tube. Peripheral blood mononuclear cells (PBMCs) were separated using a lymphocyte-separating medium, followed by centrifugation at 840 g for 10 min at 4°C using an ultrahigh-speed centrifuge to extract the upper-layer plasma. Plasma samples were stored at −80°C, and clinical information was recorded. All samples were further processed in a Biosafety level 2 laboratory-qualified for severe acute respiratory syndrome coronavirus 2 testing in accordance with the Laboratory Biosafety Guidelines for COVID-19 (2nd edition)issued by the National Health Commission of China.

In this study, plasma samples of 44 patients with COVID-19 (including 31 mild COVID-19patients and 13 severe COVID-19patients), 42 healthy volunteers, and 42 patients with community-acquired pneumonia (CAP) were collected. PBMCs samples of nine COVID-19patients (including five mild COVID-19patients and four severe COVID-19patients), five healthy volunteers, and five CAP patients were also collected. In addition, clinical information was recorded, and the sample information for all patients and healthy volunteers are presented in Table 1.

***Full-Spectrum Metabolic Identification and Data Acquisition***

Our analytical method is named “wildly-targeted metabolome”, which is similar to non-targeted metabolomics.But the advantage of our method is that we have built our own metabolite database, which included more than 3000 metabolites, and the method has beenpublished previously.[^1^](#_ENREF_1)^,^ [^2^](#_ENREF_2)

UPLC-MS/MS (ExionLC AD, QTRAP®, https://sciex.com.cn/) was used to acquiredata. The liquid-phase conditions were as follows: (1) chromatographic column using Waters ACQUITY UPLC HSS T3 C18 1.8 µm, 2.1 mm ×100 mm; (2) mobile phase with ultrapure water (0.1% formic acid) for phase A and acetonitrile (0.1% formic acid) for phase B; (3) elution gradients were water/acetonitrile (95:5 V/V) at 0 min, 10:90 V/V at 11.0 min, 10:90 V/V at 12.0 min, 95:5 V/V at 12.1 min, and 95:5 V/V at 14.0 min; (4) the flow rate was0.4 ml/min, the column temperature was 40°C, and the injection volume was 2μl. The MS conditions were as follows: 500°C for electrospray ionization, 5500 V (positive) and −4500 V (negative), 55 psi for ion gas source I (GSI), 60 psi for gas source II (GSII), 25 psi for curtain gas,andhigh collision-activated dissociation parameters. In a triple quadrupole (Qtrap), the ions were scanned and detected according to an optimized decluttering potential and collision energy. Qualitative analysis was performed based on the retention time, daughter–parent ion pair information, and secondary spectral data of the detected substances usingtheMetWare database. Quantitative analysis was accomplished using the multiple-reaction monitoring mode of Qtrap. After collecting data for different samples, the area under the peak was scored separately for the chromatographic peaks of the extracted ions of all metabolites, followed by score correction for thechromatographic peaks of the same metabolite in different specimens.[^3^](#_ENREF_3)

***Differential Metabolite EnrichmentAnalysis***

Univariate and multivariate analyses were performed to identify the differentially expressed metabolites. Data were analyzed from multiple perspectives based on their characteristics to accurately identify differentially expressed metabolites. Principal component analysis (PCA) and partial least squares-discriminant analysis (PLS-DA) were used to reduce data dimensionality and to verify the separation trends between groups. PCA and PLS-DA were performed within R(version, 4.1.1) software. To investigate the changingtrend of the relative contents of the metabolites in different samples, the relative contents of the differentially expressed metabolites were normalized and centralized, and K-mean clustering (K-means) analysis was then performed. The results of orthogonal projections to latent structures-discriminant analysis (OPLS-DA) yielded variable importance in projection (VIP) for each metabolite, and metabolites with VIP ≥ 1 were selected. In addition, metabolites with fold change ≥ 2 or ≤ 0.5 were identified as significantly differentially expressed metabolites. The potential functions of the differentially expressed metabolites were further investigated using Kyoto Encyclopedia of Genes and Genomes (KEGG) functional annotation enrichment analysis.

***Screening of potential biomarkers and model constructionfor severe COVID-19***

The least absolute shrinkage and selection operator (LASSO) regression is a penalty function that finds the most representative molecules by differentially scaling down the variables. Therefore, in this study, LASSO regression was used to screen metabolites specifically expressed in patients with severe COVID-19, and logistic stepwise regression was performed to construct a diagnostic model. In addition, receiver operating characteristic (ROC) curves were utilized to assess the ability of the model to differentiate the severe COVID-19group from the other groups.

***Whole-Transcriptome Sequencing and Data Analysis of PBMCs***

RNA extraction was performed according to the instructions of the total RNA extraction kit provided by Tiangen Biotech Co., Ltd.(Beijing). RNA concentration and purity were measured using the ND-1000 (NanoDrop Technologies, Inc., DE, USA), and RNA content was calculated as the absorbance value at 260 nm. After total RNA extraction, subsequent experiments were performed according to the standard procedure provided by Illumina (San Diego, CA, USA), including library preparation and sequencing experiments, using the Illumina Novaseq™ 6000. Small RNA sequencing libraries were prepared using the TruSeq Small RNA Sample Prep Kits (Illumina). After library preparation, the libraries were sequenced to a single-end read length of 1X50 bp. Duplex-specific libraries were constructed by depleting ribosomal RNA (rRNA depletion). The library was sequenced after passing quality control, with a double-end read length of 2X50 bp (PE150).

***Transcriptome-Metabolome Association Analysis***

The shared differentially expressed microRNAs (miRNAs)in the three groups were analyzed. The miRNA target genes were predicted by three databases, such as, miRDB, miRTarBase, and TargetScan, and the overlapping parts of the three databases were selected. In addition, Gene Ontology (GO) and KEGG enrichment analyses were performed to explore possible regulatory mechanisms. The shared differentially expressed miRNAs in the three groups were identified and subsequently subjected to KEGG enrichment analysis. Gene set variation analysis (GSVA) was performed separately in the severe COVID-19group and the other groups to further explore potential mechanisms in the pathogenesis of severe COVID-19.

***Statistical Analysis***

Experimental data are presented as mean ± standard deviation, and p < 0.05 was considered significant. Continuous parametric variables in the three groups were tested by the analysis of variance, while continuous nonparametric variables were tested using the nonparametric Mann–Whitney U test. Qualitative dataanalysis was performed using the chi-square test. The above analyses were performed with R version 4.1.1(R Foundation for Statistical Computing, Vienna, Austria).

**
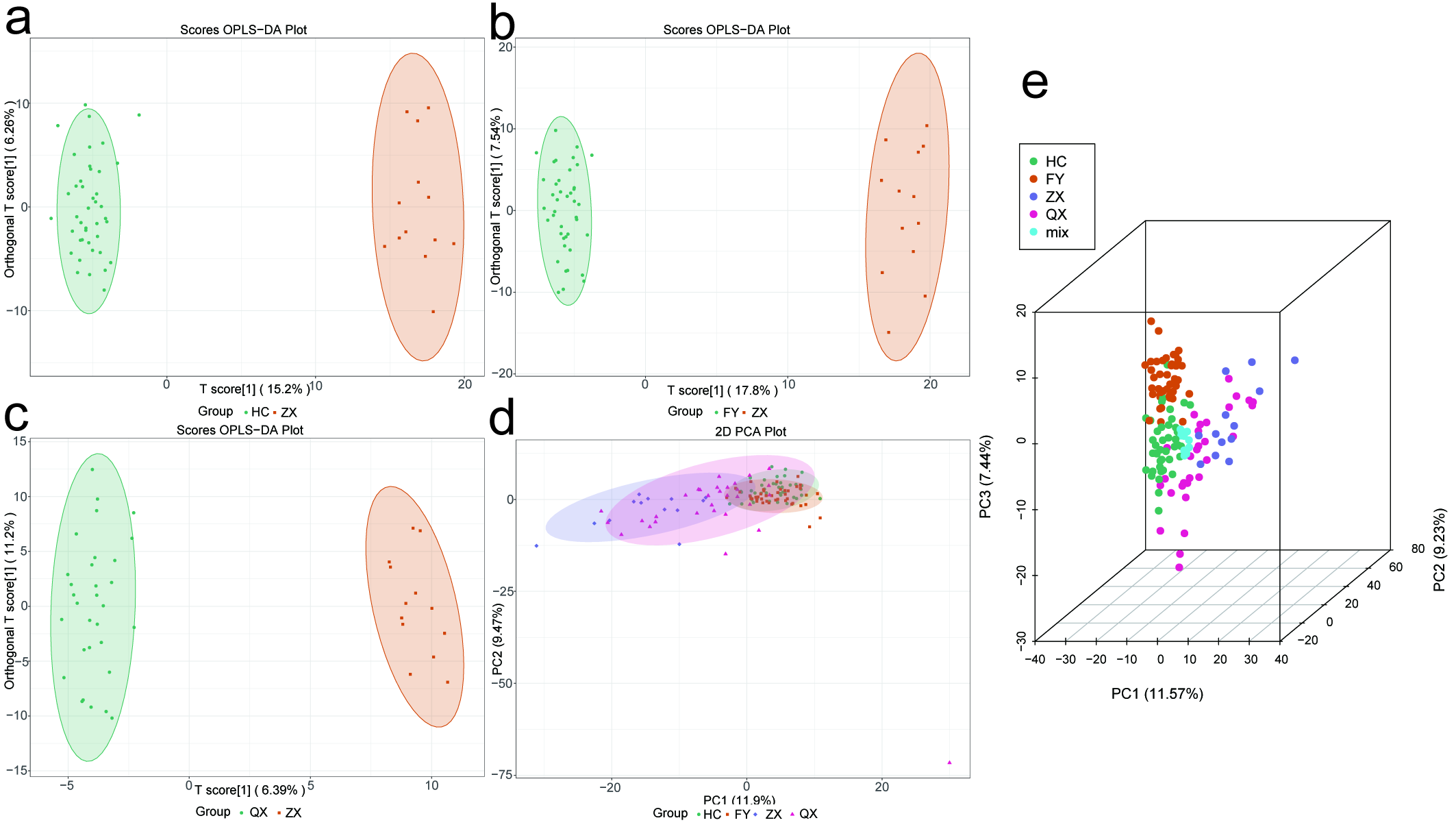
**

**Supplementary Fig. S1.** **Differences in metabolites between the four groups.** HC, healthy controls. FY, community acquired pneumonia. QX, mild COVID-19 patients. ZX, severe COVID-19 patients. Score plots by orthogonal partial least squares discriminant analysis (OPLS-DA) for severe COVID-19 patients vs. healthy controls (a), community acquired pneumonia (CAP) patients (b), and mild COVID-19patients (c). 2D principal component analysis (PCA) (d) and 3D-PCA (e) revealed the differences in metabolites between the four groups.

**
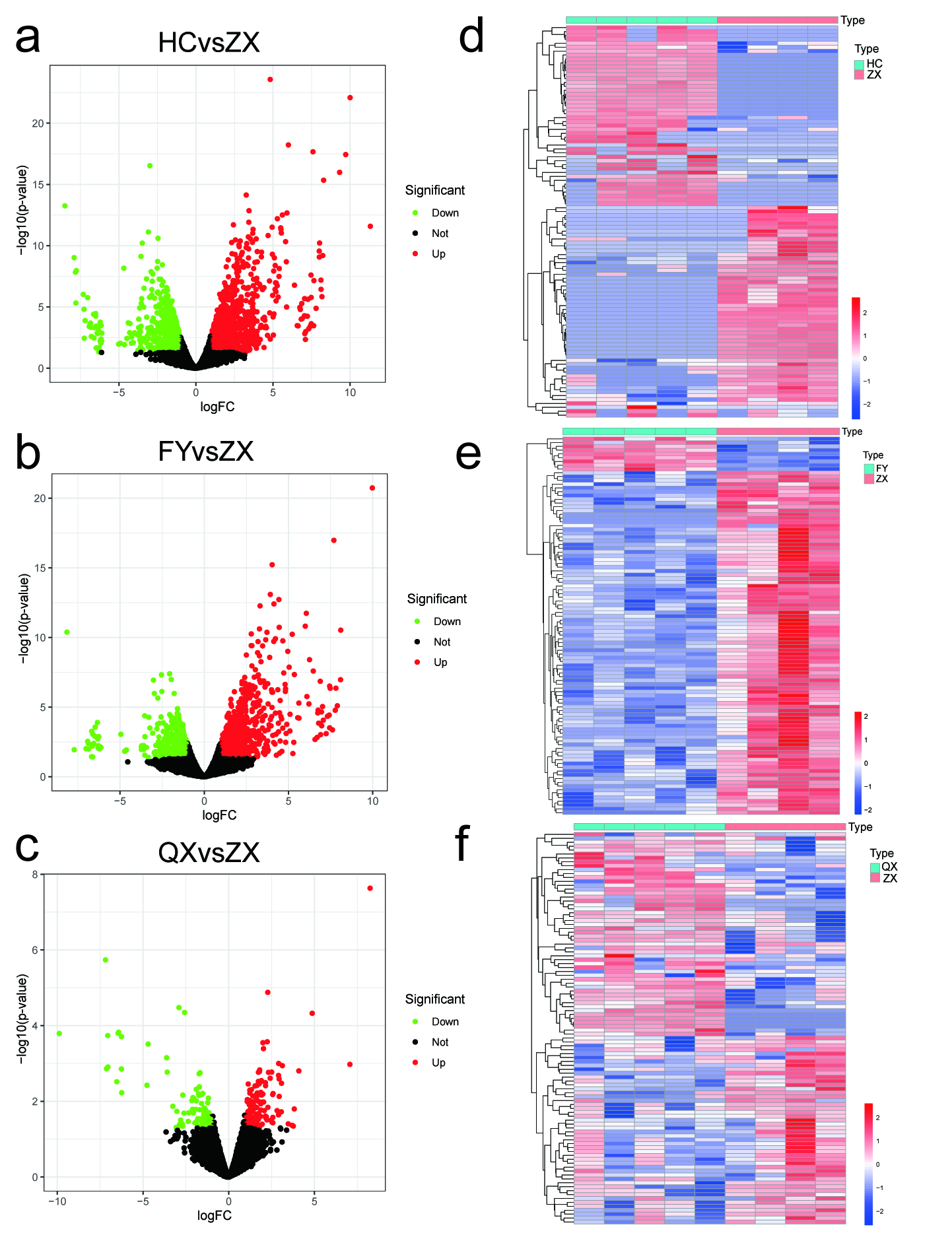
**

**Supplementary Fig. S2.** **Identification of differentially expressed lncRNAs.** HC, healthy controls. FY, community acquired pneumonia. QX, mild COVID-19 patients. ZX, severe COVID-19 patients. Volcano plot demonstrating differentially expressed lncRNAs between severe COVID-19, healthy control (a), community acquired pneumonia (CAP) (b), and mild COVID-19 (c) groups. Heat map of the expressions of differentially expressed lncRNAs between the severe COVID-19, healthy control (d), CAP (e), and mild COVID-19 (f) groups.

**
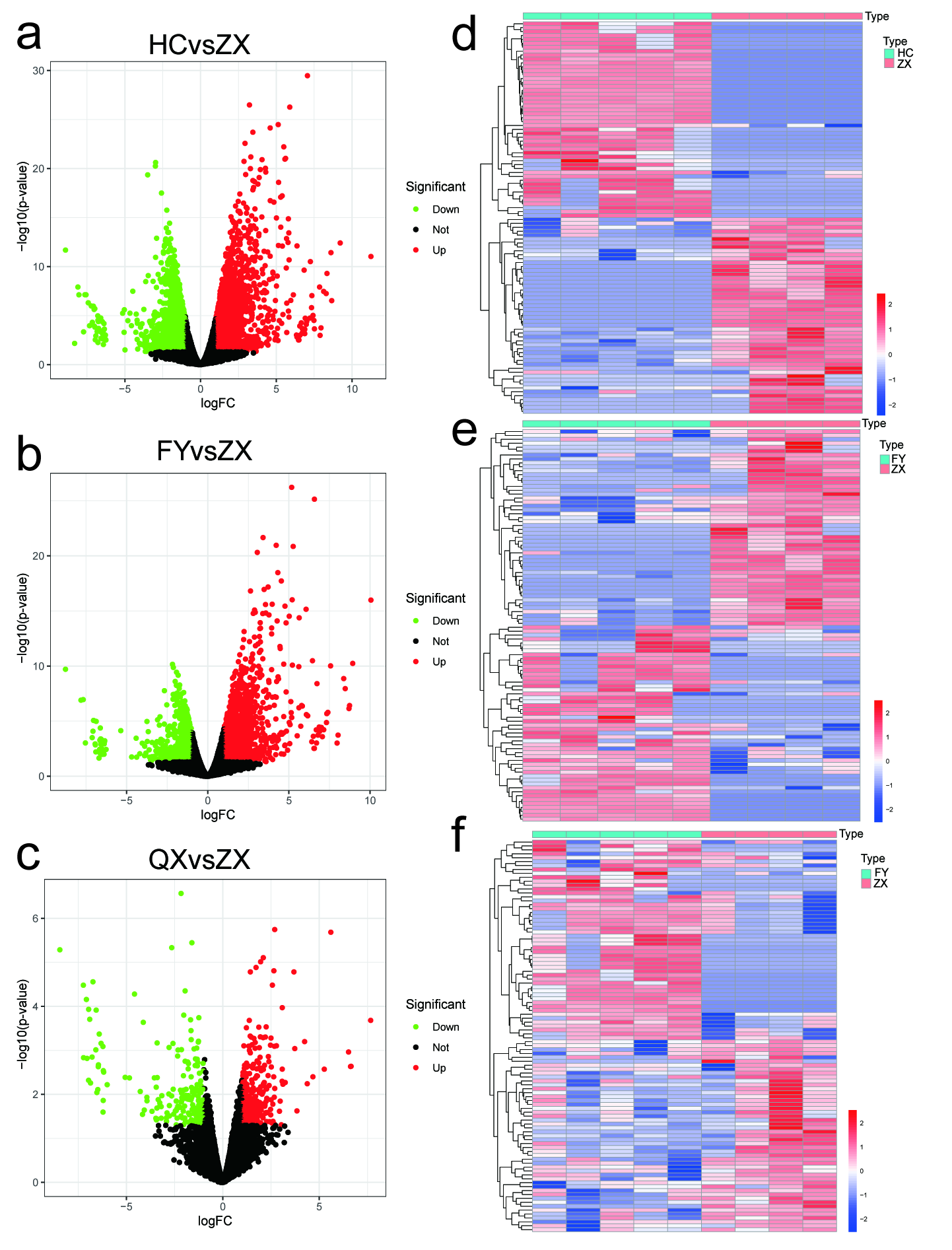
**

**Supplementary Fig. S3. Identification of differentially expressed mRNAs.** HC, healthy controls. FY, community acquired pneumonia. QX, mild COVID-19 patients. ZX, severe COVID-19 patients. Volcano plot demonstrating expressions of differentially expressed mRNAs between the severe COVID-19, healthy control (a), community acquired pneumonia (CAP) (b), and mild COVID-19 (c) groups. Heat map of the expression of differentially expressed mRNAs between the severe COVID-19, healthy control (d), CAP (e), and mild COVID-19 (f) groups.

**
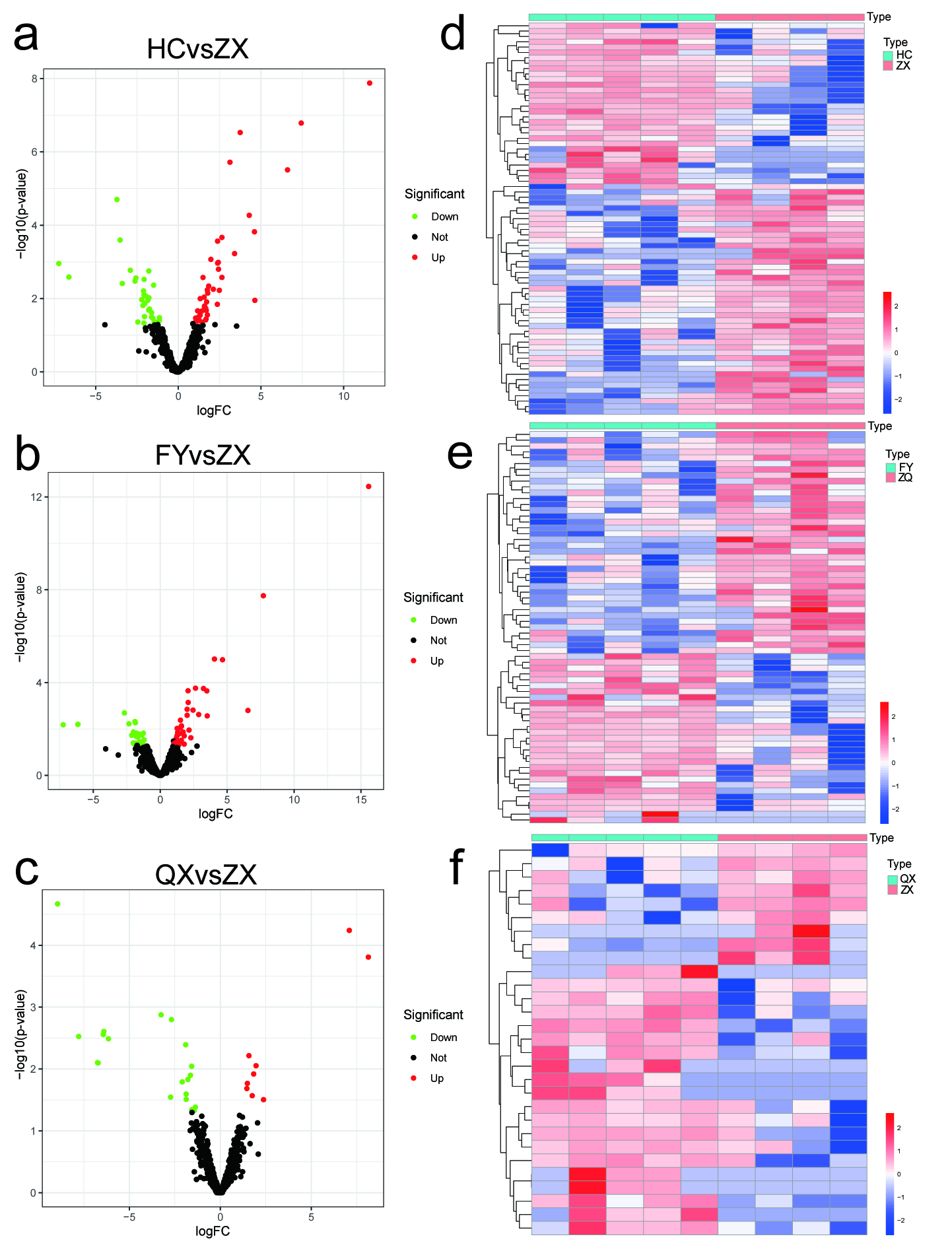
**

**Supplementary Fig. S4.** **Identification of differentially expressed circRNAs.** HC, healthy controls. FY, community acquired pneumonia. QX, mild COVID-19 patients. ZX, severe COVID-19 patients. Volcano plot demonstrating expressions of differentially expressed circRNAs between the severe COVID-19, healthy control (a), community acquired pneumonia (CAP) (b), and mild COVID-19 (c) groups. Heat map of the expression of differentially expressed circRNAs between the severe COVID-19, healthy control (d), CAP (e), and mild COVID-19 (f) groups.

**
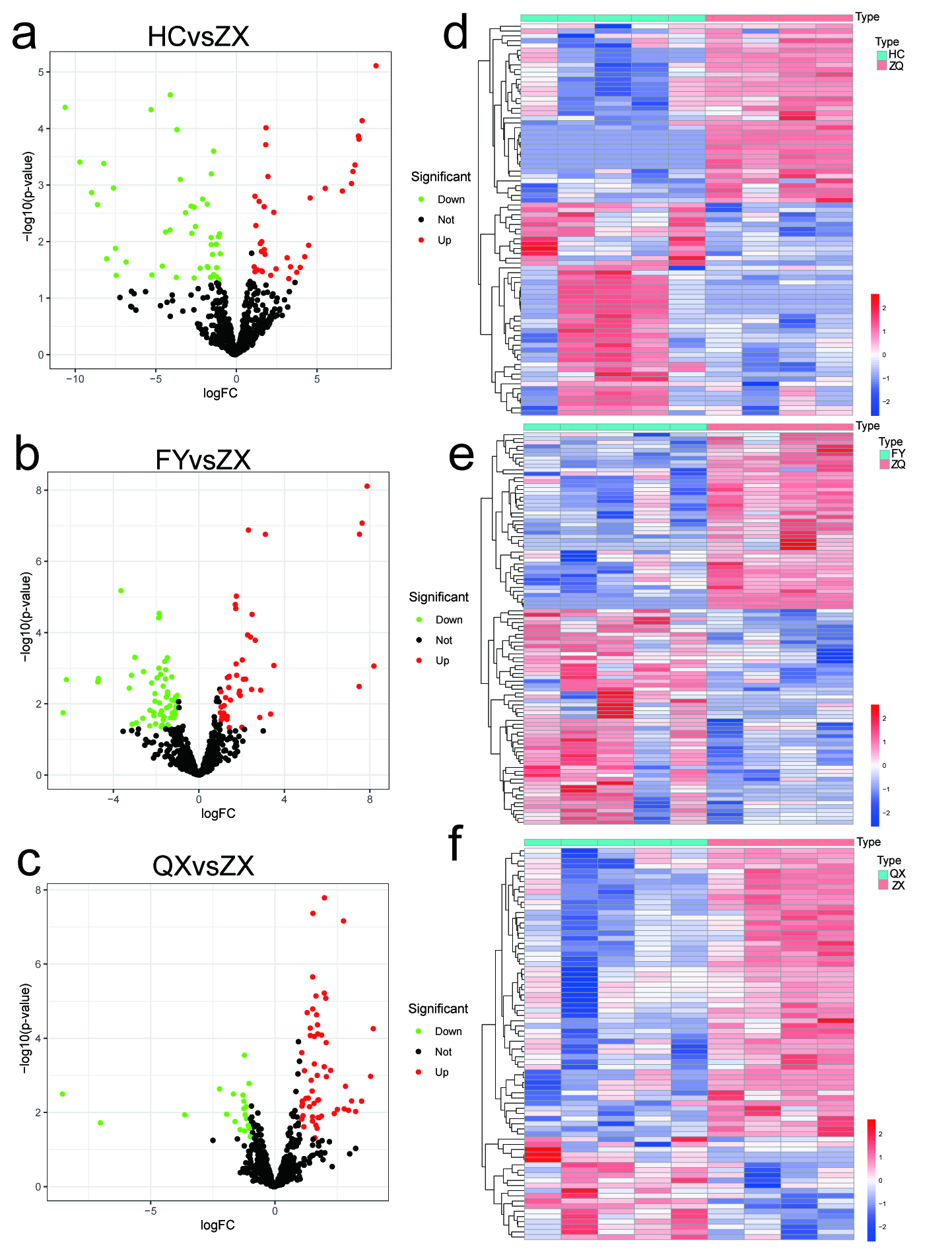
**

e

**Supplementary Fig. S5.** **Identification of differentially expressed miRNAs.** HC, healthy controls. FY, community acquired pneumonia. QX, mild COVID-19 patients. ZX, severe COVID-19 patients. Volcano plot demonstrating expressions of differentially expressed miRNAs between the severe COVID-19, healthy control (a), community acquired pneumonia (CAP) (b), and mild COVID-19 (c) groups. Heat map of the expression of differentially expressed miRNAs between the severe COVID-19, healthy control (d), CAP (e), and mild COVID-19 (f) groups.

**
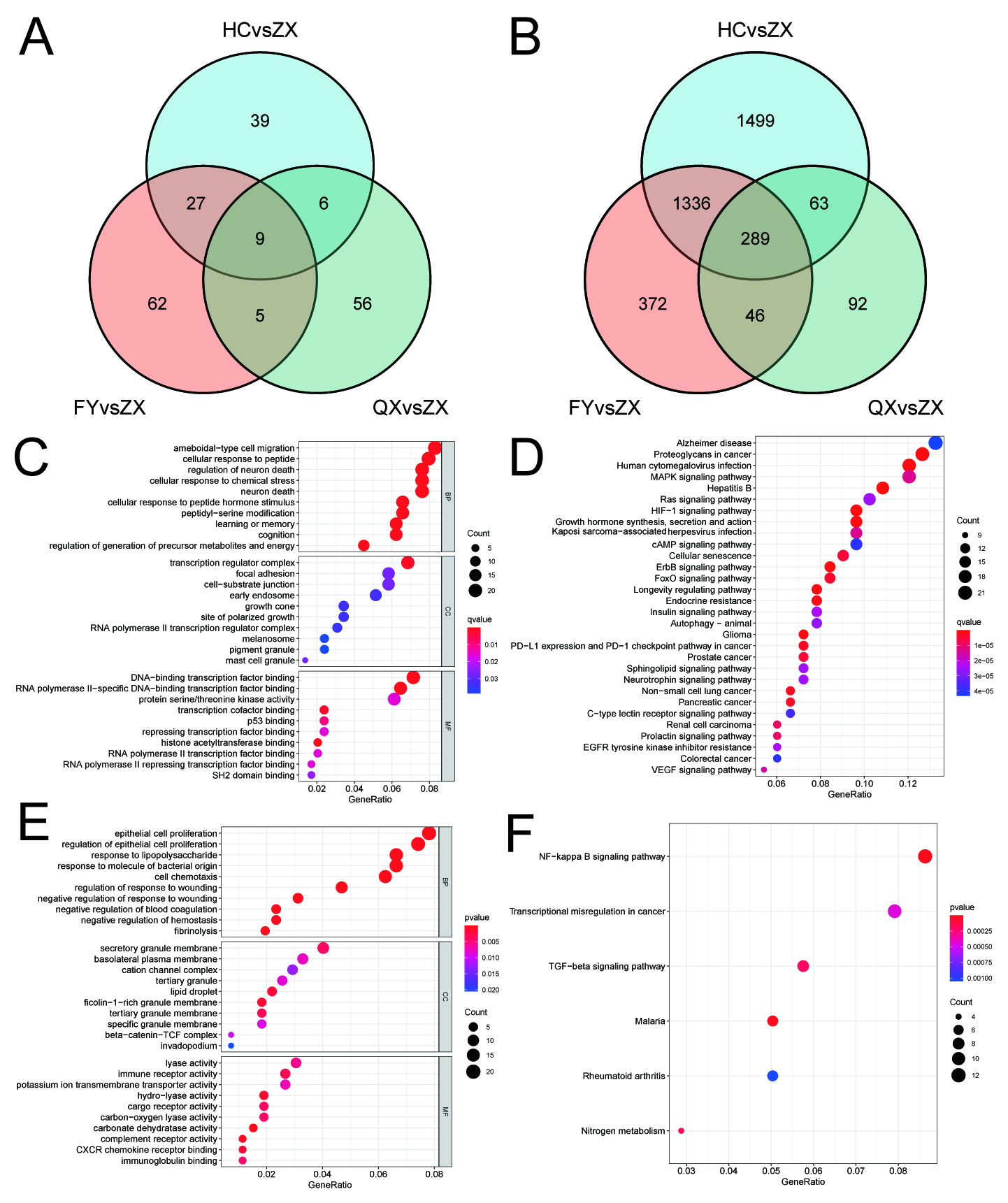
**

b

c

d

f

e

a

**Supplementary Fig. S6. Identification and enrichment analysis of differentially expressed miRNAs and mRNAs.** HC, healthy controls. FY, community acquired pneumonia. QX, mild COVID-19 patients. ZX, severe COVID-19 patients. (a) Venn diagram demonstrating miRNAs specific to severe COVID-19. (b) Venn diagram of mRNA profiles specific to severe COVID-19. (c) Gene Ontology (GO) analysis of potential target genes of miRNAs specific to severe COVID-19. (d) Kyoto Encyclopedia of Genes and Genomes (KEGG) enrichment analysis of potential target genes of miRNAs specific to severe COVID-19. GO (e) and KEGG (f) enrichment analysis of shared differentially expressed mRNA molecules.

**
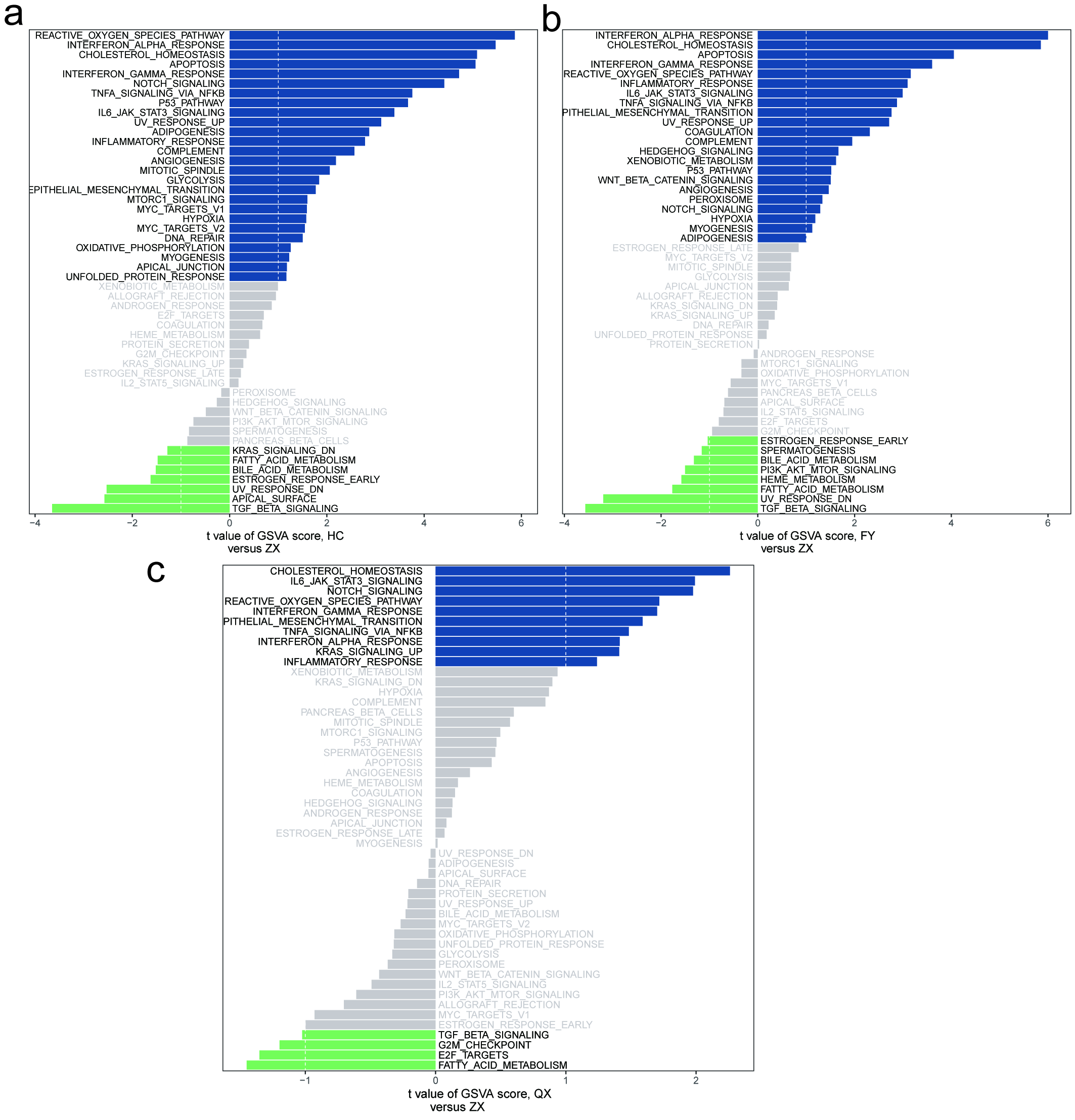
**

**Supplementary Fig. S7. Gene set variation analysis (GSVA).** HC, healthy controls. FY, community acquired pneumonia. QX, mild COVID-19 patients. ZX, severe COVID-19 patients. GSVA analysis demonstrating differential pathways in the severe COVID-19, healthy control (a), CAP (b), and mild COVID-19 (c) groups.

**Supplementary Table S1.**

**Characteristics of the groups**

| **Data type** | **Method** | **Detail** | **Sample size** | | | |
| --- | --- | --- | --- | --- | --- | --- |
|  |  |  | **severe COVID-19** | **mild COVID-19** | **CAP**  **group** | **Healthy**  **group** |
|  |  |  | **group** | **group** |  |  |
| metabolites | Full-Spectrum Metabolic Identification | Total Number | 13 | 31 | 42 | 42 |
|  |  | Gender (male/Female) | 7/6 | 16/15 | 22/20 | 22/20 |
|  |  | Ethnicity (Han Chinese) | 13 | 31 | 42 | 42 |
|  |  | Age (years, median; range) | 52(30-74) | 41(20-64) | 45.5(26-68) | 42.5(26-68) |
|  |  |  |  |  |  |  |
| mRNAs lncRNAs  circRNAs miRNAs | Whole-  Transcriptome Sequencing | Total Number | 4 | 5 | 5 | 5 |
|  |  | Gender (Male/Female) | 2/2 | 3/2 | 3/2 | 4/1 |
|  |  | Ethnicity (Han Chinese) | 4 | 5 | 5 | 5 |
|  |  | Age (years, median; range) | 33.5(30-74) | 39(33-46) | 53(33-57) | 48(34-63) |

CAP, community-acquired pneumonia.

**Supplementary Table S2.**

**Detailed information of ten metabolites in diagnostic model**

| Index | Formula | Compounds | Class I | Class II |
| --- | --- | --- | --- | --- |
| MEDN0472 | C6H6N4O3 | 1-Methyluric Acid | Amino acid and Its metabolomics | Amino acid derivatives |
| MEDP1032 | C7H6O2 | Salicylaldehyde | Benzene and substituted derivatives | Benzene and substituted derivatives |
| MEDP1409 | C19H37NO5 | Carnitine C12-OH | FA | CAR |
| MEDP1406 | C21H39NO4 | Carnitine C14:1 | FA | CAR |
| MEDN1283 | C23H42NO7P | PysoPE 18:3  (2n isomer) | GP | LPE |
| MEDN1274 | C25H46NO7P | PysoPE 20:3 | GP | LPE |
| MEDN1272 | C25H48NO7P | PysoPE 20:2 | GP | LPE |
| MEDP0576 | C12H28N4O | N1-Acetylspermine | Organic acid And Its derivatives | Organicacid and Its derivatives |
| MEDN0333 | C3H4O4 | Malonicacid | Organic acid And Its derivatives | Organicacid and Its derivatives |
| MEDN0292 | C4H8O3 | 3-Hydroxybutyrate | Organic acid And Its derivatives | Organicacid and Its derivatives |

**Supplementary Table S3**

**Characteristics of clinical data**

| **Characteristic** | **mild COVID-19**  **group** | **severe COVID-19**  **group** | **Healty**  **group** | **CAP**  **group** | **p** |
| --- | --- | --- | --- | --- | --- |
| n | 31 | 13 | 39 | 38 |  |
| Sex, n |  |  |  |  | 0.428 |
| Female | 15 | 6 | 16 | 20 |  |
| Male | 16 | 7 | 23 | 18 |  |
| Age, meidan (IQR) | 41 (35.5, 49) | 52 (35, 55) | 50 (35, 57) | 46 (37.75, 55) | 0.415 |
| TG, meidan (IQR) | 1.52 (0.91, 1.98) | 1.14 (0.84, 1.34) | 1.54 (0.97, 2.76) | 1.79 (1.29, 2.21) | 0.744 |
| CH, meidan (IQR) | 3.93 (3.44, 4.48) | 4.2 (3.31, 4.95) | 4.56 (3.09, 5.28) | 4.05 (3.16, 4.95) | 0.632 |
| HDLC, meidan (IQR) | 1.07 (0.92, 1.19) | 1.09 (0.91, 1.18) | 1.06 (0.84, 1.75) | 1.37 (1.08, 1.63) | <0.05 |
| LDLC, meidan (IQR) | 2.53 (2.22, 3.15) | 2.9 (2.29, 3.3) | 3.17 (1.94, 3.76) | 2.63 (2.16, 3.5) | 0.684 |
| ALT, meidan (IQR) | 15 (13, 28.5) | 21 (20, 34) | / | / | 0.153 |
| AST, meidan (IQR) | 22 (19, 27) | 28 (26, 33) | / | / | <0.05 |
| GGT, meidan (IQR) | 24 (17.5, 30.5) | 25 (17, 50) | / | / | 0.537 |
| TBIL, meidan (IQR) | 11.7 (7.7, 17.15) | 10.7 (7.2, 16) | / | / | 0.979 |
| DBIL, meidan (IQR) | 4 (2.5, 5.65) | 3.8 (3, 5.4) | / | / | 0.827 |
| TBA, meidan (IQR) | 4.9 (3.15, 7.1) | 3.2 (2, 7.6) | / | / | 0.643 |

CAP, community-acquired pneumonia. TG, triglyceride. CH,cholesterol. HDLC, high density cholesterol. LDLC, Low density cholesterol. ALT, Alanine aminotransferase. AST, aspartate aminotransferase. GGT, glutamyl transpeptidase. TBIL, total bilirubin. DBIL, direct bilirubin. TBA, total bile acid. IQR, interquartile range.

**Supplementary References**

1. Fu, Y. et al. Elevation of JAML Promotes Diabetic Kidney Disease by Modulating Podocyte Lipid Metabolism. *Cell Metab* **32**, 1052-1062.e1058 (2020).

2. Jing, Y. et al. SARS-CoV-2 infection causes immunodeficiency in recovered patients by downregulating CD19 expression in B cells via enhancing B-cell metabolism. *Signal transduction and targeted therapy* **6**, (2021).

3. Fraga, C.G., Clowers, B.H., Moore, R.J., Zink, E.M. Signature-discovery approach for sample matching of a nerve-agent precursor using liquid chromatography-mass spectrometry, XCMS, and chemometrics. *Analytical chemistry* **82**, 4165-4173 (2010).
