# Supplementary material for: Novel potential metabolic biomarker panel for early detection of severe COVID-19 using full-spectrum metabolome and whole-transcriptome analyses
Source: Signal Transduct Target Ther. 2022 Apr 22;7:129. doi: 10.1038/s41392-022-00976-2 (PMC9026054; doi:10.1038/s41392-022-00976-2)
Supplement: Supplementary file 2 — Sup materials2-ethic approval [file 41392_2022_976_MOESM2_ESM.pdf]

浙江大学细胞生物研究所科研项目申请伦理审批件

日期：2021 年 3 月 29 日

申请项目名称	基于多组学联合分析的 COVID-19 生物标志物研究		
研究课题来源	国家自然科学基金；省自然科学基金； 其他 √		
项目负责人	李继承	学 院	医学院
联系电话	13600511900	Email	lijichen@zju.edu.cn

审查	研究者资格：符合要求 √ 不符合要求□
	获取知情同意书方式：适当 √ 不适当□
	试验方案：适当 √ 不适当□
研究目的	研究将筛选新冠肺炎患者血液中差异表达的生物标志物。在筛选生物标志物的基础上，通过多组学关联与整合分析，研究旨在寻找新冠肺炎生物标志物，为临床研究奠定基础。
研究内容(附研究方案)	采用代谢组学技术筛选普通、重症新冠患者及社区获得性肺炎、健康对照组血液中差异表达的代谢物。通过 KEGG 功能富集分析，预测这些差异代谢物参与的主要代谢通路。借助 LASSO 回归和 logistics 回归，构建出新冠肺炎，以及重症新冠肺炎的有效诊断模型。同时，本研究将采用全转录组测序技术筛选差异表达的 RNA，并进行代谢组-转录组关联分析，为新冠肺炎的病理机制研究提供新的线索。

审批结论	同意	同意 稍作修改	修改后再次会议讨论	不同意
	√			

审批意见	经研究所医学伦理委员会审议，认为“基于多组学联合分析的 COVID-19 生物标志物研究”研究方案和知情同意书符合伦理学原则。同意研究项目实施。<br>盖章：浙江大学 细胞生物学研究所
